# Mobilization practices in critically ill children: a European point prevalence study (EU PARK-PICU)

**DOI:** 10.1186/s13054-020-02988-2

**Published:** 2020-06-24

**Authors:** Erwin Ista, Barnaby R. Scholefield, Joseph C. Manning, Irene Harth, Orsola Gawronski, Alicja Bartkowska-Śniatkowska, Anne-Sylvie Ramelet, Sapna R. Kudchadkar, EU PARK-PICU Collaborators, Paul C. Ritson, Filippia Nikolaou, Marjorie de Neef, Martin Kneyber, Kate Penny-Thomas, Christina Linton, Reinis Balmaks, Matthias Richter, Fabrizio Chiusolo, Corrado Cecchetti, Marco Roberti, Michela Di Furia, Chantal Grandjean, Bettina Nygaard, Yolanda Lopez, Tolga Koroglu, Tolga Besci, Roberta Da Rin Della Mora, Rachel S. Agbeko, Emma Borrows, Nathalie Bochaton, Janet Mattsson, Anne Ksellmann, Barbara Hero, Jowita Rosada-Kurasinska, Magdalena Świder, Amabile Bonaldi, Cristina Giugni, Siva Oruganti, Simon Gates, Hazel Smith, Annelies van Zwol, Jenna Hills, Johanna Conroy, Mark Bebbington, Felix Neunhoeffer, Els Duval

**Affiliations:** 1 Pediatric Intensive Care Unit, Department of Paediatric Surgery, Erasmus Medical Center – Sophia Children’s Hospital, P.O. Box 2060, 3000 CB Rotterdam, The Netherlands; 2 Nursing Science, Department of Internal Medicine, Erasmus Medical Center, Rotterdam, The Netherlands; 3 Birmingham Acute Care Research Group, Institute of Inflammation and Ageing, University of Birmingham, Birmingham, UK; 4 Paediatric Intensive Care Unit, Birmingham Women & Children’s Hospital, Birmingham, UK; 5 Nottingham Children’s Hospital & Neonatology, Nottingham University Hospitals NHS Trust, Nottingham, UK; 6 School of Health Sciences, The University of Nottingham, Nottingham, UK; 7 Pediatric Intensive Care Unit, Universitätsmedizin Mainz, Zentrum für Kinder- und Jugendmedizin, Mainz, Germany; 8 Healthcare professional development Unit, Bambino Gesù Children’s Hospital, Rome, Italy; 9 Department of Pediatric Anesthesiology and Intensive Therapy, Poznań University of Medical Sciences, Poznań, Poland; 10 Institute of Higher Education and Research in Healthcare, University of Lausanne and Lausanne University Hospital, Lausanne, Switzerland; 11 Department of Anesthesiology and Critical Care Medicine, Johns Hopkins University School of Medicine, Baltimore, MD USA; 12 Department of Pediatrics, Johns Hopkins University School of Medicine, Baltimore, MD USA; 13 Department of Physical Medicine & Rehabilitation, Johns Hopkins University School of Medicine, Baltimore, MD USA; 14 Alder Hey Children’s NHS Foundation Trust, Liverpool, UK; 15 Children Hospital ‘P & A ‘Aglaia Kyriakou, Athens, Greece; 16 Amsterdam UMC-Emma Children’s Hospital, Amsterdam, The Netherlands; 17 University Medical Center Groningen, Beatrix Children’s Hospital, Groningen, The Netherlands; 18 Birmingham Women & Children’s Hospital, Birmingham, UK; 19 Bristol Royal Hospital for Children, Bristol, UK; 20 Children’s Clinical University Hospital, Riga, Latvia; 21 Universitätsklinikum Carl Gustav Carus Klinik für Kinder- und Jugendmedizin, Dresden, Germany; 22 Children’s Hospital Bambino Gesù – PICU, Rome, Italy; 23 Children’s Hospital Bambino Gesù - Emergency Department PICU, Rome, Italy; 24 Children’s Hospital Bambino Gesù –CICU, Rome, Italy; 25 Children’s Hospital Bambino Gesù, Palidoro, Italy; 26 Lausanne University Hospital (CHUV), Lausanne, Switzerland; 27 Copenhagen University Hospital, Rigshospitalet, Copenhagen, Denmark; 28 Cruces University Hospital, Bilbao, Spain; 29 Dokuz Eylül University, Izmir, Turkey; 30 IRCCS Istituto Giannina Gaslini, Genova, Italy; 31 Great North Children’s Hospital, Newcastle upon Tyne, UK; 32 Great Ormond Street Hospital, London, UK; 33 Geneva University Hospital (HUG), Geneva, Switzerland; 34 Karolinska, Stockholm, Sweden; 35 Kinderherzzentrum Sankt Augustin, St Augustin, Germany; 36 Children’s University Hospital Cologne, Köln, Germany; 37 Kliniczny Szpital Wojewódzki No 2 w Rzeszowie, Rzeszów, Poland; 38 Ospedale Maggiore, Verona, Italy; 39 Meyer Children’s Hospital, Florence, Italy; 40 Noah’s Ark Children’s Hospital for Wales, Cardiff, UK; 41 Nottingham Children’s Hospital and Neonatology, Nottingham University Hospitals NHS Trust, Nottingham, England; 42 Children’s Health Ireland at Crumlin, Dublin, Ireland; 43 Radboudumc-Amila Children’s Hospital, Nijmegen, The Netherlands; 44 Royal Hospital for Children, Glasgow, Scotland; 45 Royal Manchester Children’s Hospital, Manchester, England; 46 Royal Stoke University Hospital, Stoke-on-Trent, UK; 47 Department of Pediatric Cardiology, Pulmonology and Pediatric Intensive Care Medicine, University Children’s Hospital Tübingen, Tübingen, Germany; 48 UZA Antwerp, Edegem, Belgium

**Keywords:** Critical care, Paediatrics, Rehabilitation, Physical therapy, Occupational therapy, Developmental paediatrics, Intensive care units

## Abstract

**Background:**

Early mobilization of adults receiving intensive care improves health outcomes, yet little is known about mobilization practices in paediatric intensive care units (PICUs). We aimed to determine the prevalence of and factors associated with physical rehabilitation in PICUs across Europe.

**Methods:**

A 2-day, cross-sectional, multicentre point prevalence study was conducted in May and November 2018. The primary outcome was the prevalence of physical therapy (PT)- or occupational therapy (OT)-provided mobility. Clinical data and data on patient mobility, potential mobility safety events, and mobilization barriers were prospectively collected in patients admitted for ≥72 h.

**Results:**

Data of 456 children admitted to one of 38 participating PICUs from 15 European countries were collected (456 patient days); 70% were under 3 years of age. The point prevalence of PT- and/or OT-provided mobility activities was 39% (179/456) (95% CI 34.7–43.9%) during the patient days, with significant differences between European regions. Nurses were involved in 72% (924/1283) of the mobility events; in the remaining 28%, PT/OT, physicians, family members, or other professionals were involved. Of the factors studied, family presence was most strongly positively associated with out-of-bed mobilization (aOR 7.83, 95% CI 3.09–19.79). Invasive mechanical ventilation with an endotracheal tube was negatively associated with out-of-bed mobility (aOR 0.28, 95% CI 0.12–0.68). Patients were completely immobile on 25% (115/456) of patient days. Barriers to mobilization were reported on 38% of patient days. The most common reported patient-related barriers were cardiovascular instability (*n* = 47, 10%), oversedation (*n* = 39, 9%), and medical contraindication (*n* = 37, 8%). Potential safety events occurred in 6% of all documented mobilization events.

**Conclusion:**

Therapists are infrequently consulted for mobilization of critically ill children in European PICUs. This study highlights the need for a systematic and interdisciplinary mobilization approach for critically ill children.

**Graphical abstract:**

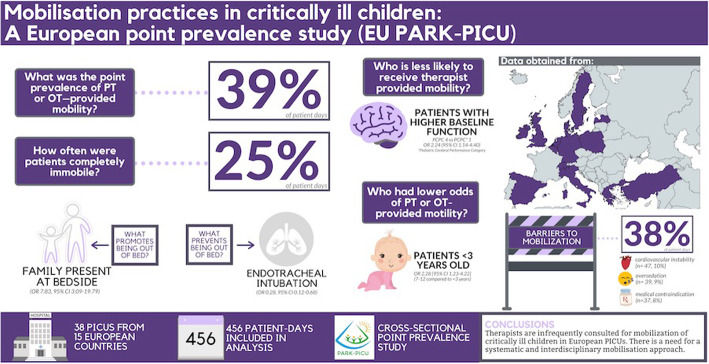

## Background

The paediatric intensive care unit is a stressful environment for critically ill children. To provide safety, comfort, and hemodynamic stability, children are often sedated and considered too sick to be mobilized [1]. However, immobility is associated with adverse effects such as muscle weakness [2, 3], pressure ulcers [4, 5], increased risk of a delirium [6], and post-intensive care syndrome [7–9]. Ideally, sedation is targeted to facilitate ICU procedures while keeping the patient comfortable without anxiety and agitation.

Early mobilization of patients in adult ICUs has proven feasible and safe with favourable clinical outcomes including decreased delirium incidence and duration of ICU stay, less sedatives consumption, and improved muscle strength and functional status [6, 10]. Early mobilization in critically ill children undergoing active neurocognitive and physical development is understudied. An early mobilization project in the USA targeting critically ill children showed promising results with no adverse events [11]; this interdisciplinary approach doubled the number of mobilization events per patient.

A recent point prevalence study of physical rehabilitation in United States PICUs (PARK-PICU: Prevalence of Acute Rehabilitation for Kids in the PICU) demonstrated that younger children and those with higher baseline function were less likely to receive therapist-provided mobility [12]. In view of the lack of similar data in European countries, we adapted the PARK-PICU study in Europe to determine the prevalence of routine mobilization of children admitted to a PICU for at least 72 h. Additionally, we explored patient-related barriers and potential adverse events related to PICU mobilization.

## Methods

The European Prevalence of Acute Rehabilitation for Kids in the PICU study (EU-PARK-PICU) was a 2-day cross-sectional point prevalence study (May 29, 2018, and November 6, 2018). PICUs from European countries were eligible to participate if they met the following criteria: (1) provide care for mechanically ventilated infants and children and (2) located in a distinct physical space within a hospital dedicated to paediatric patients. Sites were recruited via the European Society of Paediatric and Neonatal Intensive Care (ESPNIC) network, e-mail, social media, and a dedicated website (https://park.web.jhu.edu). Institutional review board or clinical governance approval was obtained at all sites with waiver of informed consent. For comparability across studies internationally, we utilized the same methodology as the original PARK-PICU study in the United States of America (USA) [12].

### Patient selection

Eligible patients were those who had been admitted to PICUs for ≥ 72 h as of 7 a.m. on each of the two study days. This criterion was set because admission longer than 3 days carries greater risk for muscle atrophy and physical impairment [13], and adult ICU studies suggest this as the threshold for early rehabilitation and mobilization [6, 14, 15].

### Notification, study day selection, and data collection

An information brochure (available in English, Italian, French, German, Polish, Spanish, and Dutch) was provided to interested sites. Each participating site was informed about the designated months for the point prevalence once consent was provided. On the first day of the month, at random a weekday for the study was chosen to conduct the study in all PICUs. The local study teams were notified by email that they should screen for eligibility on that day and prepare for data collection on the following day, starting at 9 a.m. and continued until 9 a.m. the next day.

### Measures

All data collection forms were adapted from those used in the USA PARK-PICU study. In addition to English, bedside data collection forms (e.g. Activity events) were translated to Dutch, French, Spain, German, and Italian are available on the study website [16].

### PICU characteristics

Each participating PICU completed an electronic online survey (LimeSurvey©) to provide administrative data and information about clinical resources and protocols related to pain, sedation and delirium management, ventilator weaning, early mobilization, and family engagement. To ensure accuracy, site investigators were instructed to complete the questionnaire together with a physician and the PICU nurse manager.

### Patient clinical characteristics

Sites extracted clinical data for all eligible patients, including clinical status at 9 a.m. on the point prevalence day: mechanical ventilation status, sedative infusions and level of sedation, delirium screening, and invasive catheters. Mechanical ventilation was defined as ventilation through an endotracheal tube, a tracheostomy, or a face/nasal mask. Pre-admission physical function categorized by the Paediatric Cerebral Performance Score (PCPC) was extracted from the medical record or, if missing, on the basis of information from family and care team [16, 17].

#### Mobility data

Physical therapy (PT) and occupational therapy (OT) consultation and treatment documentation for the first 72 h of PICU admission were abstracted from the electronic patient record or other records. Standardized forms with checklists were distributed to the bedside of each eligible patient by 9 a.m. for real-time event recording. Nurses and other healthcare staff were instructed to document the following: (a) occurrence of any mobility activity provided by a therapist, nurse, family, and/or other staff; (b) type (in-bed/out-of-bed) and timing of mobility events; (c) perceived barriers to mobilization; and (d) safety events associated with mobilization—e.g. a change of ≥ 10% in heart rate, oxygen saturation, or blood pressure; loss of invasive devices; and falls. Out-of-bed mobility was defined as transfer from bed to chair, being held by family or staff, mat play, standing, or walking. Activities such as passive motion, sitting in bed, and bath were defined as in-bed mobility. Mobility events were defined as any single activity or clustered activities involving the child’s physical movement, with the exception of routine care procedures and turning/repositioning or prevention of pressure ulcers. Each separate activity was recorded on a separate form. Both barriers and potential safety events were selected from a pre-specified list with a free-text option.

The primary outcome was “therapist-provided mobility”, defined as at least one mobility event performed by a PT or OT on the study day. This primary outcome measure was chosen for the following reasons: (1) comparability to US paediatric and adult point prevalence data [18] and (2) information from participating PICUs that PT and OTs are often consulted simultaneously and the rehabilitation team determines which services are most appropriate to provide. Secondary outcomes were out-of-bed mobility, barriers to mobilization, and potential safety events.

### Data analysis

The prevalence of therapist-provided mobility was defined as the number of patient days with therapist-provided mobility divided by the total number of patient days across the two study days. Also, the prevalence of out-of-bed mobility was calculated as the number of patients with out-of-bed mobility provided by a healthcare professional (e.g. PT, OT, or nurse) divided by the total number of patient days across the study days. Data of patients discharged before 12 p.m. on the study days were excluded from the final analysis. Categorical data were analysed using Fisher’s exact test or chi-squared test and are expressed as frequency. Continuous data were analysed using the Kruskal-Wallis test and are expressed as median (IQR). Multivariable regression models (generalized estimating equations), with a random effect for site, were used to evaluate variables associated with therapist-provided mobility events and out-of-bed mobility (see Additional file 1). Age (< 3 or ≥ 3 years) was explored as statistical interaction with covariates for the outcome of out-of-bed mobility. Adjusted odds ratios (aOR) are presented with 95% confidence interval (CI). For the purpose of analysis, sites were categorized by European region using the definition in the end-of-life practices in intensive care units study (see Additional file 1, [19]. Two-tailed *P* < 0.05 was considered statistically significant. IBM SPSS version 25.0 was used for all statistical analyses.

## Results

### ICU characteristics

A total of 38 PICUs from 15 countries participated, representing all European regions (see Additional file 1 - eFigure1). Most were located in northern Europe (47%) and were part of an academic teaching hospital (76%), and half were paediatric medical-surgical-cardiac units (Additional file 1, eTable 1). The number of beds ranged from 4 to 31, with a median of 12 (IQR 9–17). Early mobilization protocols were available in 16% of the units. Dedicated PT or OT staff were present in 61% and 18% of the PICUs, respectively. In 87% (33/38), a prescription of a physician and/or nurse was required to consult a PT and/or OT.

### Patient baseline characteristics

In total, 722 patients were screened for eligibility; 456 (63%) patients were included (Additional file 1, eFigure 1). Fifty-three percent were boys, 70% were under the age of 3 years, and the median PICU length of stay on the study day was 14 days (IQR 6–36) (Table 1). Most were medical patients (62%), and 59% of all included patients had surgery during the PICU admission. For 47% of all included patients, the PCPC prior to admission was > 2, indicative of moderate or severe neurological/cognitive disability.

**Table 1:** Patient baseline characteristics

Characteristics	Patients	PT/OT-provided mobility	No PT/OT-provided mobility	***p*** value
	***N*** = 456	***N*** = 179	***N*** = 277	
**Gender**, female, *n* (%)	213 (46.7)	88 (49.2)	125 (45.1)	0.442
**Age,** years, *n* (%)				0.033
0–2	319 (70.0)	111 (62.0)	208 (75.1)	
3–6	39 (8.6)	17 (9.5)	22 (7.9)	
7–12	54 (11.8)	27 (15.1)	27 (9.7)	
13–18	41 (9.0)	23 (12.8)	18 (6.5)	
> 18	3 (0.6)	1 (0.6)	2 (0.7)	
**Ethnicity**, *n* (%)				0.3
White	360 (78.9)	140 (78.2)	220 (79.4)	
Black	27 (5.9)	8 (4.5)	19 (6.9)	
Asian	43 (9.4)	17 (9.5)	26 (9.4)	
Hispanic	4 (0.8)	2 (1.1)	2 (0.7)	
Other	22 (4.8)	12 (6.7)	10 (3.6)	
**Baseline PCPC**, *n* (%)				0.031
1: Good	107 (23.5)	37 (20.7)	70 (25.3)	
2: Mild disability	124 (26.1)	37 (20.7)	82 (29.6)	
3: Moderate disability	96 (21.1)	43 (24.0)	53 (19.1)	
4: Severe	127 (27.9)	58 (32.4)	69 (24.9)	
5: Coma/vegetative state	7 (1.5)	4 (2.2)	3 (1.1)	
**Reason of admission**, *n* (%)				0.013
*Medical*				
Respiratory	125 (27.4)	49 (27.4)	76 (27.4)	
Cardiac	61 (13.4)	17 (9.5)	44 (15.9)	
Haematology/oncology	16 (3.5)	8 (4.5)	8 (2.9)	
Infectious/inflammatory	21 (4.6)	13 (7.3)	8 (2.9)	
Neurologic	36 (7.9)	13 (7.3)	23 (8.3)	
Other	26 (5.7)	10 (5.6)	16 (5.8)	
*Post-surgical*				
Cardiac	81 (17.8)	27 (15.1)	54 (19.5)	
Neuro	19 (4.2)	14 (7.8)	5 (1.8)	
Ortho	3 (0.7)	1 (0.6)	2 (0.7)	
Other	68 (14.9)	27 (15.1)	41 (14.8)	
**Source of admission**, *n* (%)				0.167
Emergency room	55 (12.1)	17 (9.5)	38 (13.7)	
Floor/step down unit	119 (26.1)	53 (29.6)	66 (23.8)	
Outside hospital	155 (34.0)	56 (31.3)	99 (35.7)	
Operation room/post-anaesthesia	69 (15.1)	34 (19.0)	35 (12.6)	
NICU	39 (8.6)	11 (6.1)	28 (10.1)	
Home	8 (1.8)	2 (1.1)	6 (2.2)	
Post-birth/delivery room	9 (1.8)	4 (2.2)	4 (1.4)	
Other	4 (0.9)	2 (1.1)	1 (0.4)	
**BMI**^b*^	15.1 (12.8–17.9)	15.6 (13.3–18.5)	14.7 (12.3–17.6)	0.002
Days of hospital stay at study day*	22 (IQR 8–60)	28 (11–80)	20 (7–57)	0.002
Days of PICU stay at study day*	14 (6–36)	17 (8–45)	12 (6–32)	0.002
Postoperative day*^1^	11 (4–31)	18 (7–39)	8 (4–22)	< 0.001
**Surgery during PICU stay**, yes (%)	278 (58.9)	107 (59.8)	164 (59.2)	0.848
**Ambulatory prior to admission if age** ≥ 3 years, no. (%)^a^	63 (46.0)	34 (50.0)	29 (42.0)	0.393
**Unit mobility protocol**, yes (%)	60 (13.2)	25 (14.0)	35 (12.6)	0.673

*PCPC* paediatric cerebral performance category, *PT* physical therapist, *OT* occupational therapist

*Median (IQR)

^1^From most recent surgery

^a^Based number of patients > 2 years

^b^Missing data: BMI (*n* = 22)

### Patient clinical characteristics

Two hundred and thirty-seven patients (52%) were mechanically ventilated through an endotracheal tube or a tracheostomy during at the time of observation (Table 2). Half of the patients received at least one continuous sedative or analgesic infusion, mostly opioids (58%) and benzodiazepines (43%). The level of sedation was assessed in 73% (174/237) of the mechanically ventilated patients. Of the ventilated patients, 75% (177/237) of the patients had a central venous catheter (CVC) in place, and 51% (120/237) of these patients had a urinary catheter.

**Table 2:** Clinical characteristics of patients by PT/OT-provided mobility

Characteristics	Patients	PT/OT-provided mobility	No PT/OT-provided mobility	***p*** value
	***N*** = 456	***N*** = 179	***N*** = 277	
**Respiratory support,*n*(%)**				0.445
No support	67 (14.7)	28 (15.6)	39 (14.1)	
Nasal cannula or face mask	30 (6.6)	12 (6.7)	18 (6.5)	
Heated high-flow nasal cannula	50 (11.0)	15 (8.4)	35 (12.6)	
Trach collar	18 (3.9)	5 (2.8)	13 (4.7)	
Non-invasive ventilation	54 (11.8)	19 (10.6)	35 (12.6)	
Mechanical ventilation via ETT	178 (39.0)	71 (39.7)	107 (38.6)	
Mechanical ventilation via tracheostomy	59 (12.9)	29 (16.2)	30 (10.8)	
FiO2*	30 (25–45)	35 (25–45)	30 (25–40)	0.091
Day of MV*	11 (5–30)	13 (6–37)	10 (5–29)	0.096
GCS*	14 (9–15)	13 (9–15)	14 (9–15)	0.470
**Sedation score documented**^a^, yes (%)	174 (73.4)	79 (79.0)	95 (69.3)	0.097
**Sedatives/analgesics: ≥ 1 continuous infusion**, *n* (%)	232 (50.9)	96 (53.6)	136 (49.1)	0.388
**Vasoactive infusions, at least one**, *n* (%)^b^	50 (11.0)	19 (10.6)	31 (11.2)	0.879
**Delirium screening performed**				0.403
No, not available	394 (86.4)	152 (84.9)	242 (87.4)	
Yes, positive screening	5 (1.1)	1 (0.6)	4 (1.4)	
Yes, negative screening	57 (12.5)	26 (14.5)	31 (11.2)	
**Restraint**, at least one, *n* (%)	61 (13.4)	21 (11.7)	40 (14.4)	0.482
**Devices**, *n* (%)				
Endotracheal tube	180 (39.5)	72 (40.0)	108 (39.0)	0.792
Tracheal cannula	81 (17.8)	37 (20.7)	44 (15.9)	0.192
Central venous catheter	290 (63.6)	118 (65.9)	172 (62.1)	0.427
Arterial line	178 (39.0)	73 (40.8)	105 (37.9)	0.556
Haemodialysis catheter, *n* (%)	21 (4.6)	7 (3.9)	14 (5.1)	0.652
ECMO cannula, *n* (%)	14 (3.1)	4 (2.2)	10 (3.6)	0.580
Foley catheter, *n* (%)	167 (36.6)	55 (30.7)	112 (40.4)	0.037
Surgical drain, *n* (%)	40 (8.8)	14 (7.8)	26 (9.4)	0.614
Chest tube, *n* (%)	39 (8.3)	14 (7.8)	25 (9.0)	0.773
Ventricular assist device, *n* (%)	11 (2.4)	3 (1.7)	8 (2.9)	0.543
Intracranial pressure monitor, *n* (%)	19 (4.2)	6 (3.4)	13 (4.7)	0.539
**Pressure ulcer(s)**, at least one, *n* (%)	48 (10.5)	15 (8.4)	33 (11.9)	0.275
**Nurse to patient ratio**, *n* (%)				0.503
2:1 or 1:1	215 (47.1)	88 (49.2)	127 (45.8)	
1:2 or 1:3	241 (52.9)	91 (50.8)	150 (54.2)	
**Family present at bedside**	354 (77.6)	160 (89.4)	194 (70.0)	< 0.001

*PT* physical therapist, *OT* occupational therapist, *HFNC* high-flow nasal cannula, *CPAP* continuous positive airway pressure, *MV* mechanical ventilation, *ETT* endotracheal tube, *ECMO* extracorporeal membrane oxygenation, *CVC* central venous catheter, *FiO2* fractional inspired oxygen concentration, *GCS* Glasgow Coma Scale

*Median (IQR)

^a^If mechanically ventilated (total, *n* = 237; PT/OT provided, *n* = 100; no PT/OT provided, *n* = 137)

^b^Excluding milrinone

### Therapist-provided consultation and mobility

The prevalence of PT- and/or OT-provided mobility was 39% (95% CI 34.7–43.9%) across the two study days. The prevalence significantly differed between the PICUs in the three European regions: northern, 36%; central, 63%; and southern, 25% (*p* < 0.001). By 72 h of PICU admission, 24% of patients had received a PT and/or OT consultation, and 21% of those received mobilization therapy. Still, 43% of patients, with a median PICU stay of 11 days (IQR 6–31), never received a PT or OT consultation. Receiving a PT and/or OT consultation was irrespective of the PCPC score (PCPC ≤ 2 22% vs. PCPC ≥ 3 26%, *p* = 0.331; Additional file 1, eFigure 3). Tables 1 and 2 display univariate analysis for demographic and clinical factors and the primary outcome of PT or OT provided mobility. We found that patients ≥ 3 years of age received more frequently therapist-provided mobility compared with age < 3 years, 35% (111/319) versus 50% (68/137) respectively (*p* = 0.003). Further, mechanically ventilated patients did not receive significantly more frequent therapist-provided mobility compared with those who were not (42% vs. 36%; *p* = 0.181). In total, 174 patient days included PT-provided mobility, and only ten patient days included OT-provided mobility. Therefore, we were not able to provide discipline-specific mobility associations.

In a logistic multivariable analysis adjusting for relevant demographic and clinical characteristics, therapist-provided mobility was associated with older age (aOR compared to age < 3: 7–12 years, 2.28 [95% CI 1.23–4.22]) and moderate and severe baseline disability (PCPC 3 vs. PCPC 1: 2.12 [95% CI 1.02–4.56] and PCPC 4 vs. PCPC1: 2.24 [95% CI 1.14–4.40]). Therapist-provided mobility was also associated with having a CVC in place (aOR 1.63; 95% CI 1.02–2.62) and with family presence during one of the study days (aOR 5.13; 95% CI 2.55–10.32). In contrast, fewer therapist-provided mobility episodes were provided to children with urinary catheter in place (aOR 0.46; 95% CI 0.22–0.92) (Additional file 1, eTable 2).

### All mobility events

**Fig. 1:**
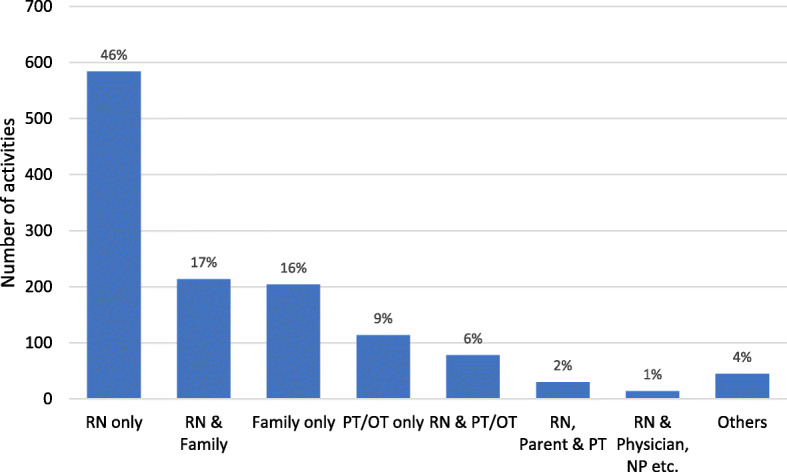
Number of activities by clinician type. RN, registered nurse; PT, physical therapist; OT, occupational therapist; NP, nurse practitioner

Mobilization events did not occur on 115 of 456 patient days (25%), notably not in mechanically ventilated patients (75 of 115 patient days, 65%). On the other 341 patient days, 1283 total mobility events occurred, with a median of 3 (IQR 3–5) per patient. In most cases (*n* = 584, 46%), a nurse alone provided the mobilization event; in 17% of cases, a nurse together with family; in 6% of cases, a nurse together with a PT or OT; and in 16% of cases, family alone (Fig. 1). There were slight differences in mobilization between the European regions (Additional file 1, eFigure 4). Nurses mobilized critically ill children in the northern European countries less frequently (40%) than in the southern (54%) and central (51%) countries.

**Fig. 2:**
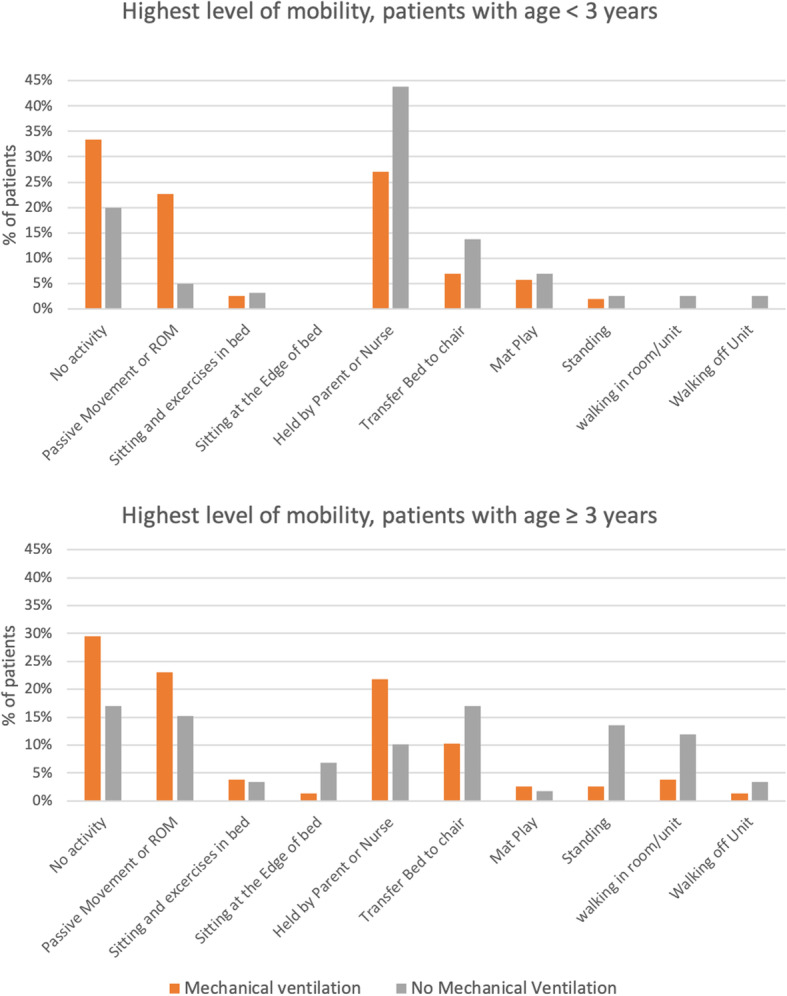
Highest level of mobility

Figure 2 shows the highest level of mobility achieved by patients on the study days. Among mechanically ventilated children, passive range of motion (age < 3, 23%; age ≥ 3, 23%) and being held by family or nurse (age < 3, 27%; age ≥ 3, 22%) were the most common mobility events. Among non-ventilated patients, being held by family or nurse was most frequent in children below age of 3 years (44%), while bed-to-chair transfer was the commonest in over 2-year-olds (17%). Children < 3 years were held by family or a nurse in 55% of central, 35% in northern, and 15% in southern European PICU (Additional file 1, eFigure 5).

#### Out-of-bed mobility

Out-of-bed mobility was achieved as the highest level of mobility for PICU patients on 248 study days, i.e. an overall prevalence of 46% (95% CI 41.0–50.3%). Seventy-eight percent of the children below 3 years of age received out-of-bed mobility compared to 22% of patients 3 years of age and older (*p* < 0.001). Non-mechanically ventilated patients were significantly more likely than mechanically ventilated patients to achieve out-of-bed mobility (70% vs. 30%; *p* < 0.001). The most common out-of-bed activity for all patients was being held by a parent or a nurse (*n* = 519), with bed-to-chair transfer without standing (*n* = 120), and mat play (*n* = 51).

#### Factors associated with out-of-bed mobility

**Fig. 3:**
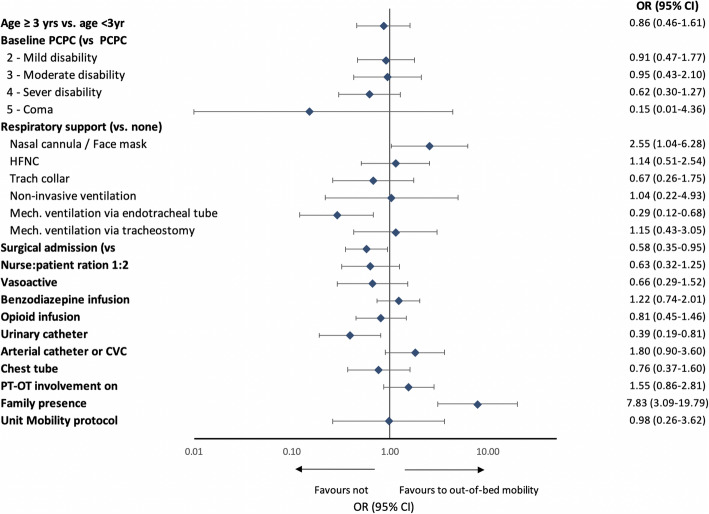
Adjusted odds ratio for out-of-bed mobility on study day. The multivariable model included random effect for site, adjusted for admission reason, gender, and ethnicity in addition to all characteristics listed. Vasoactive infusion excluded milrinone. HFNC, high-flow nasal cannula; CPAP, continuous positive airway pressure; MV, mechanical ventilation; ETT, endotracheal tube; PCPC, paediatric cerebral performance category; CVC, central venous catheter; PT, physical therapist; OT, occupational therapist; EM, early mobilization

In a multivariable logistic regression model (Fig. 3, and Additional file 1, eTable 3), mechanical ventilation through an endotracheal tube (aOR 0.29, 95% CI 0.12–0.68), being admitted for a surgical reason (aOR 0.58, 95% CI 0.35–0.95), and the presence of a urinary catheter (aOR 0.39, 95% CI 0.19–0.81) were negatively associated with out-of-bed mobility. Family presence had a strong positive association with out-of-bed mobility (aOR 7.83, 95% CI 3.09–19.79).

### Perceived barriers to mobilization

At least one barrier to mobilization was reported for 177 of 456 patients (39%), with two or more barriers reported for 87 of these 177 patients (Additional file 1, eTable 4). Barriers were reported for 66% (76/115) of the patient days without mobilization activities. Barriers to mobilization were reported significantly more frequently for mechanically ventilated patient than for non-ventilated patients (49% vs. 28%; *p* < 0.001). The most common barriers were cardiovascular instability (*n* = 47, 10%), oversedation (*n* = 39, 9%), and medical contraindication (*n* = 37, 8%).

### Potential adverse events

Staff reported potential safety events in 74 (6%) of 1286 mobility events in 43 patient days (9%). These were more commonly among mechanically ventilated compared to non-ventilated patients (8% vs. 5%; *p* = 0.026). The most commonly reported potential safety events were decreased oxygen saturation (*n* = 33, 3%), change in heart rate (*n* = 25, 2%), and change in blood pressure (*n* = 20, 2%). An endotracheal tube dislocation occurred only once. There were no cardiac arrests or falls reported.

## Discussion

This study presents the first estimates of routine mobilization practices in European PICUs. Mobilization did not occur on 25% of the study days, and when mobility did occur, it was facilitated mostly by nurses alone. Older children and children with severe disability most frequently received mobilization interventions provided by a physical therapist or occupational therapist with the help of nurses. One quarter of the patients were not mobilized at all due to cardiac instability, oversedation, or medical contraindication. We found that the rate of potential safety adverse events (6%) was low relative to the large number of mobility events and similar to that reported in paediatric (4%) and adult (3%) studies [20]. Most of these potential safety events were transient vital sign changes. Only 0.2% of all mobility events were associated with dislodgement of a device, comparable to the 0.6% rate in adults [20]. Therefore, our data, in parallel with evidence from single-centre PICU studies [21, 22], suggest that mobilization of PICU patients is safe.

There are several important similarities and differences with the USA-PARK-PICU findings [12], a retrospective Canadian multicentre PICU study [23], and adult point prevalence studies of ICU mobilization practices [18]. Our observation of a 39% prevalence of therapist-provided mobility on the study days is consistent with the USA data (35%) and the Canadian study (30%) [23]. However, there was a large difference between the central European and northern and southern countries. A lower proportion of EU-PICUs (16%) reported a unit-based mobility or rehabilitation protocol compared to the USA (27%). The minority of units with systematic mobility approaches may in part explain the moderate prevalence of mobilization interventions provided by a physical therapist or occupational therapist. Integrating PTs and OTs into routine PICU care is integral to advancing infant and toddler’s cognitive and physical development, especially important given that 70% of all PICU patients were < 3 years of age [24, 25]. Notably, mechanically ventilated children were less likely to receive therapist-provided mobilization, in line with point prevalence studies in adults but different from USA-PARK-PICU where there was no significant difference [18, 26–28].

Our study highlights the crucial role of nurses in mobilization of critically ill children. Nurses are a constant presence at the bedside, so it is not surprising that they provided the majority of mobilization events. In both the USA-PARK-PICU study and USA ARDS-Net point prevalence study in adults, nurses facilitated two thirds of mobility events [12, 18]. PICU nurses understand the importance of early mobilization but may consider it as additional workload which may translate to lower prioritization [29]. A strong collaboration with therapists, unit-based protocols in place, and facilities and equipment for mobilization could facilitate the implementation of early mobilization in daily practice [30]. Although it would be a challenge for many PICUs with nursing shortages to accommodate such changes in practice, there may be a great benefit to patients with shorter PICU stays and duration of mechanical ventilation, less delirium, and reduced costs as has been observed in adults [31].

Presence of family was very strongly associated with increased out-of-bed mobility in our study. While the USA-PARK-PICU study observed that family presence was positively associated with mobility in children under 3, our European data magnifies this association powerfully. It is likely these family members provided assistance to facilitation success with the mobilization procedure. Parents indeed have a uniquely supportive role during physiotherapy that clinicians cannot provide [32, 33] which can help to decrease the child’s anxiety and increase buy-in to participate [34]. With family visitation restrictions heightened during a global pandemic such as COVID-19, strategies are urgently needed to ensure that mobility is not negatively impacted.

Professional and organizational issues such as time constraints, lack of resources to implement early mobilization, and factors related with invasive devices and patient characteristics could be barriers to early rehabilitation across all ICU populations [35]. Our study show that decreased out-of-bed mobility of patients with an endotracheal tube and invasive devices in place is consistent with finding from adult studies and USA-PICU data. However, a unique finding of the present study, similar to the USA, was that an indwelling urinary catheter was a barrier to out-of-bed mobility. Daily review of the potential for devices to be removed can both reduce the risk of hospital-related infections and avoid confining patients to bed. Out-of-bed mobility can be safe, when a device is secured during the pre-mobility planning [36], especially if a dedicated multi-professional mobilization protocol and trained team are available. Other perceived barriers to out-of-bed mobility included medical status, lack of physician order, isolation precautions, and oversedation. Having a mobilization protocol in place would, however, not be sufficient to overcome all these kinds of barriers. A culture change among PICU team is warranted.

Our study has several limitations. First, only PICUs with sufficient research staffing or interest in early mobilization may have participated, potentially biasing the results to overestimate mobilization practices. Still, this risk is low because only 16% of the participating PICUs had a formal protocol. Second, mobility assessments were unblinded, which may have led to greater mobility delivery on the study days. Efforts had been made to limit knowledge of the study, and the observed relatively low prevalence makes it unlikely that single-day escalation of mobility efforts has biased the overall estimates. Third, we were not able to report whether a patient was medically able to be mobilized or get out of bed. Fourth, combining PT- and OT-provided mobility as the primary outcome may not recognize their unique contributions. But, due to small numbers of OT-provided mobility, we were not able to provide discipline-specific mobility associations. Further, in lack of knowing the proportion of PTs/OTs who work exclusively in the PICU, we could not analyse the difference in therapist-provided mobility between those who work exclusively in the PICU and those who not. Finally, data of non-participating PICUs were not available, thereby limiting the generalizability of the results. Although a remarkable number of 38 PICUs from 16 European countries was involved in this study, the number of participating PICUs per country differed greatly, with the strongest representation of PICUs from Northern Europe. In view of the regional differences, the results of this study should be interpreted with caution. Further, France, one of the biggest European countries with many PICUs, was not represented in this study. There may be additional cultural differences in PICU practices across Europe, and the study findings cannot be considered a true representation of all PICUs in Europe.

## Conclusions

In this point prevalence study, 25% of critically ill children across Europe were completely immobile. When mobilization occurred, the most common activity was children being held by family or nurses. Nurses are the most frequent providers of mobilization and therapists are less frequently consulted, bringing to light differences between European regions and highlighting the need for a systematic, interprofessional approach to mobilization across PICUs. Removing modifiable barriers such as oversedation and lack of medical order, combined with facilitation of parental involvement, will be important to increase mobilization and rehabilitation in European PICUs, especially in ventilated children. Finally, the short- and long-term impacts of early mobilization and rehabilitation programmes should be evaluated in critically ill children to determine best practice for paediatric critical care.

## Supplementary information

**Additional file 1.** Mobilisation practices in critically ill children: A European point prevalence study (EU PARK-PICU), contain the following information: 1 Additional description of data analysis. 2 eTables. 3 eFigures.

## Data Availability

The datasets used and/or analysed during the current study are available from the corresponding author on reasonable request.

## Abbreviations

PICU: Paediatric intensive care unit
PCPC: Paediatric Cerebral Performance Score
PT: Physical therapy
OT: Occupational therapy
