# Supplementary material for: Mobilization practices in critically ill children: a European point prevalence study (EU PARK-PICU)
Source: Crit Care. 2020 Jun 24;24:368. doi: 10.1186/s13054-020-02988-2 (PMC7311184; doi:10.1186/s13054-020-02988-2)
Supplement: Supplementary file 1 — Additional file 1. Mobilisation practices in critically ill children: A European point prevalence study (EU PARK-PICU), contain the following information: 1 Additional description of data analysis. 2 eTables. 3 eFigures. [file 13054_2020_2988_MOESM1_ESM.pdf]

Additional File 1 - Mobilisation practices in critically ill children: A European point prevalence study (EU PARK-PICU)

Content

1 Additional description of data analysis 1

2 eTables 2

3 eFigures 6

1 Additional description of data analysis

Countries were divided into 3 geographic regions prior to data analysis: northern (Denmark, Ireland, Latvia, the Netherlands, Sweden, and United Kingdom), central (Belgium, Czechia, Germany, Poland, Switzerland), and southern (Greece, Israel, Italy, Spain, and Turkey). [18]

The regression model for out-of-bed mobility included these variables in addition to a binary indicator of therapist-provided mobility on the study day. Covariates in the regression analysis had been identified *a priori* on the basis of expected clinical relevance and prior literature findings. We have checked for collinearity of covariates and linear association of outcomes with continuous variables. The regression model for therapist-provided mobility included age category, sex, baseline PCPC before hospital admission, medical vs. surgical admission, type of respiratory support, vasoactive, opioid, and benzodiazepine infusions, nurse:patient ratio, indwelling urinary catheter, central venous or arterial catheters, unit mobility protocol, and family presence.

2 eTables

eTable 1 **Characteristics of participating PICUs (n=38)**

**Characteristic**	n (%)
PICU by European Region	
Northern	18 (47.4)
Central	10 (26.3)
Southern	10 (26.3)
Hospital type	
Academic teaching hospital	29 (76.3)
Free standing children's hospital	8 (21.1)
Community hospital	1 (2.6)
Type of PICU	
Medical	6 (15.8)
Medical / Surgical	12 (31.6)
Medical / Surgical/Cardiac	19 (50.0)
Cardiac	1 (2.6)
Number of beds	
1-10 beds	16 (42.1)
11-19 beds	15 (39.5)
20-29	6 (15.8)
30 or more	1 (2.6)
Median number of beds (IQR)	12 (9-17)
ICU private rooms, all	4 (10.5)
Protocols	
Sedation weaning protocol (yes)	25 (65.8)
Nurse driven	2 (5.3)
Physician driven	23 (60.5)
Ventilator weaning protocol (yes)	20 (52.6)
Nurse driven	6 (15.8)
Physician driven	14 (36.8)
Early mobilization protocol (yes)	6 (15.8)

eTable 2 Multivariable Adjusted Odds Ratios for Therapist-Provided Mobility

**Characteristics**	**All (N=456)**	
	**Adjusted OR [b] (95% CI)**	**p-value**
**Age**, years		
- 0-2	1 (reference)	
- 3-6	1.63 (0.68-3.88)	0.27
- 7-12	2.28 (1.23-4.22)	0.009
- 13-18	2.67 (0.98-7.27)	0.055
- >18	2.49 (0.55-11.21)	0.23
**Baseline PCPC**		
- No	1 (reference)	
- Mild disability	1.19 (0.58-2.43)	0.64
- Moderate disability	2.12 (1.02-4.56)	**0.046**
- Severe	2.24 (1.14-4.40)	**0.02**
- Coma/Vegetative state	5.12 (0.74-35.53)	0.099
**Respiratory support**		
- No support	1 (reference)	
- Nasal cannula or face mask	0.85 (0.30-2.38)	0.75
- Heated HFNC	0.65 (0.33-1.27)	0.21
- Trach collar	0.48 (0.12-1.92)	0.31
- Non-invasive ventilation	0.63 (0.36-1.10)	0.11
- Mechanical ventilation - ETT	0.88 (0.47-1.63)	0.68
- Mechanical ventilation - tracheostomy	1.29 (0.65-2.57)	0.47
**Surgical admission (vs medical)**	0.67 (0.40-1.13)	0.13
**Nurse:patient ration 1:2**	0.91 (0.44-1.87)	0.79
**Any vasoactive infusion (except milirone)**	1.12 (0.62-2.03)	0.71
**Benzodiazepine infusion**	0.82 (0.44-1.54)	0.53
**Opioid infusion**	1.23 (0.77-1.97)	0.39
**Urinary catheter**	0.46 (0.22-0.92)	**0.027**
**CVC**	1.63 (1.02-2.61)	**0.043**
**Arterial catheter**	1.38 (0.73-2.62)	0.33
**Chest tube**	0.75 (0.25-2.27)	0.62
**Family presence**	5.13 (2.55-10.32)	**<0.001**
**Unit Mobility protocol**	1.11 (0.56-2.22)	0.76

eTable 3 Univariate and Multivariable Adjusted Odds Ratios for Out-of-Bed Mobility

**Characteristics**	**All (N=456)**			
	**Crude Odd Ratio [a] (95% CI)**	**p-value**	**Adjusted OR [b] (95% CI)**	**p-value**
**Age**, years				
- < 3	1 (reference)		1 (reference)	
- ≥ 3	1.13 (0.65-1.94)	0.67	0.86 (0.46-1.61)	0.64
**Baseline PCPC**				
- No	1 (reference)		1 (reference)	
- Mild disability	1.11 (0.56-2.20)	0.76	0.91 (0.47-1.77)	0.78
- Moderate disability	1.07 (0.51-2.23)	0.87	0.95 (0.43-2.10)	0.91
- Severe	1.64 (0.84-3.23)	0.15	0.62 (0.30-1.27)	0.19
- Coma/Vegetative state	NA		0.15 (0.01-4.36)	0.27
**Respiratory support**				
- No support	1 (reference)		1 (reference)	
- Nasal cannula or face mask	0.40 (0.11-1.47)	0.17	2.55 (1.04-6.28)	**0.042**
- Heated HFNC	0.88 (0.33-2.37)	0.80	1.14 (0.51-2.54)	0.76
- Trach collar	1.47 (0.60-3.62)	0.40	1.04 (0.22-4.93)	0.42
- Non-invasive ventilation	0.99 (0.25-3.88)	0.99	0.67 (0.26-1.75)	0.96
- Mechanical ventilation - ETT	3.53 (1.50-8.31)	0.004	0.29 (0.12-0.68)	**0.005**
- Mechanical ventilation - tracheostomy	0.88 (0.35-2.25)	0.79	1.15 (0.43-3.05)	0.78
**Surgical admission (vs medical)**	1.74 (1.02-2.97)	0.04	0.58 (0.35-0.95)	**0.03**
**Nurse:patient ration 1:2**	1.55 (0.91-2.65)	0.11	0.63 (0.32-1.25)	0.19
**Any vasoactive infusion (except milirone)**	1.49 (0.65-3.45)	0.34	0.66 (0.29-1.52)	0.33
**Benzodiazepine infusion**	0.82 (0.44-1.53)	0.53	1.22 (0.74-2.01)	0.43
**Opioid infusion**	1.23(0.63-2.39)	0.55	0.81 (0.45-1.46)	0.48
**Urinary catheter**	2.51 (1.43-4.41)	0.001	0.39 (0.19-0.81)	**0.012**
**Arterial catheter or CVC**	0.56 (0.31-1.01)	0.06	1.80 (0.90-3.60)	0.09
**Chest tube**	1.29 (0.56-3.01)	0.54	0.76 (0.37-1.60)	0.46
**PT-OT involvement on study day**	0.64 (0.39-1.05)	0.08	1.55 (0.86-2.81)	0.15
**Family presence**	0.13 (0.07-0.25)	<0.001	7.83 (3.09-19.79)	**<0.001**
**Unit Mobility protocol**	1.02 (0.31-1.01)	0.94	0.98 (0.26-3.62)	0.97

Legend:
Covariates in the regression analysis had been identified a priori on the basis of expected clinical relevance and prior literature findings.

[a]Crude estimates include random effect for site and no adjustment for any covariate.
[b]Adjusted estimate includes random effect for site, in addition to adjustment for gender and ethnicity and all variables listed in the table.

PT – physical therapist, OT – occupational therapist, PCPC – pediatric cerebral performance category, HFNC – High Flow Nasal Cannula, CPAP – continuous positive airway pressure, MV – Mechanical ventilation ETT – endotracheal tube, CVC - central venous catheter

eTable 4 Frequency of reported barriers to mobilization

**Type of barrier**	**N (%)**
- Instability of heart or circulation*	47 (10.3)
- Over sedation*	39 (8.6)
- Medical contraindication*	37 (8.1)
- Postoperative restrictions*	23 (5.0)
- No physician order*	21 (4.6)
- Mechanical ventilation	14 (3.1)
- Bleeding risk*	13 (2.9)
- Large catheter*	12 (2.6)
- Contradiction from the patient *	10 (2.2)
- Pain*	10 (2.2)
- ECMO*	9 (2.0)
- Patient not present (off the unit for a procedure / imaging) *	7 (1.5)
- Previously immobile (patient was immobile prior to admission) *	7 (1.5)
- Acquired neuromuscular weakness *	6 (1.3)
- Cerebral (epilepsy, EEG)	5 (1.1)
- Agitation	4 (0.9)
- Critical airway	4 (0.9)
- Terminal stage*	4 (0.9)
- Contradiction from the parents*	3 (0.7)
- Fracture (risk)	3 (0.7)
- Lack of necessary equipment*	3 (0.7)
- No nursing staff available*	3 (0.7)
- Scheduling (patient unavailable when therapist was)	3 (0.7)
- Bipap for intubated	2 (0.4)
- Delirium*	2 (0.4)
- Devices (drains, evds, chest tubes etc.)	2 (0.4)
- Isolation precautions*	2 (0.4)
- ICP	2 (0.4)
- chemical paralysis	1 (0.2)
- No therapist available*	1 (0.2)
- Obesity	1 (0.2)
- Desaturation	-

* pre-specified option for survey item, “If there were more mobilisation barriers, please select all the barriers to mobilisation”.

3 eFigures

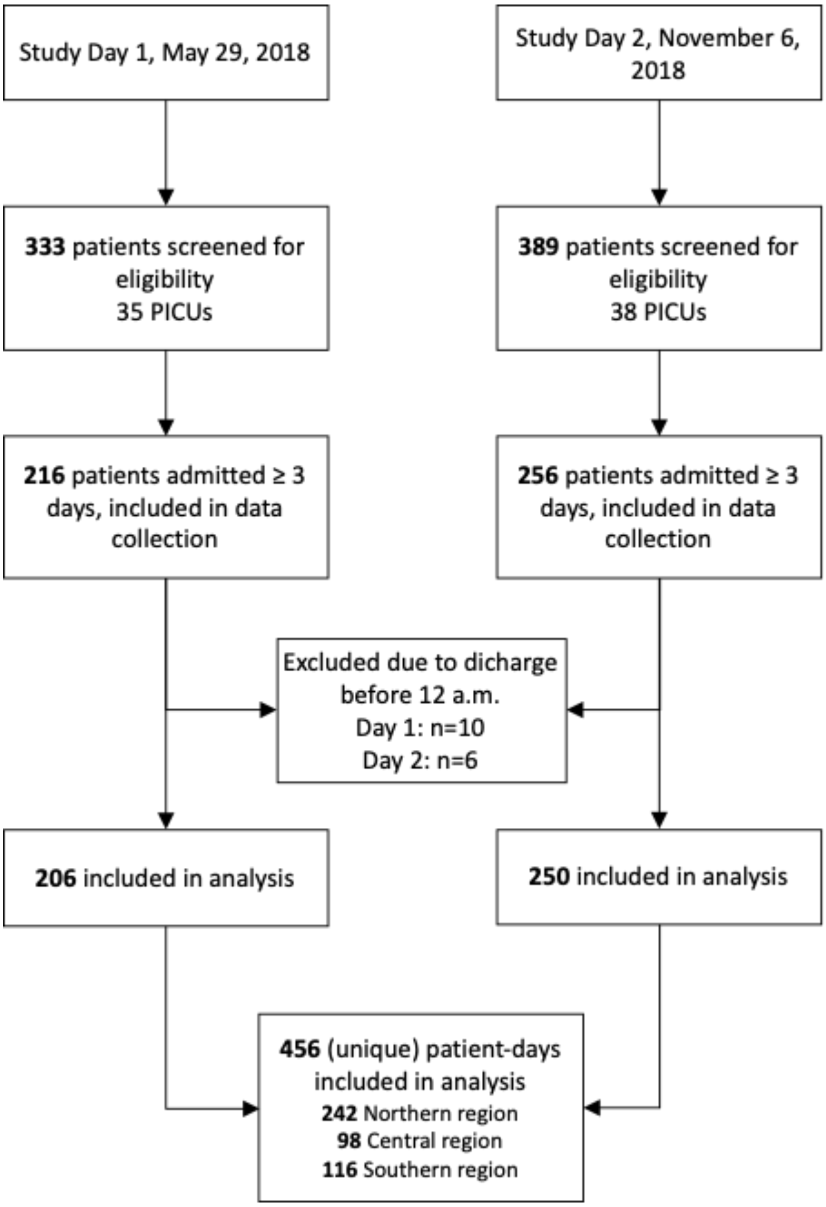

eFigure 1 Flowchart

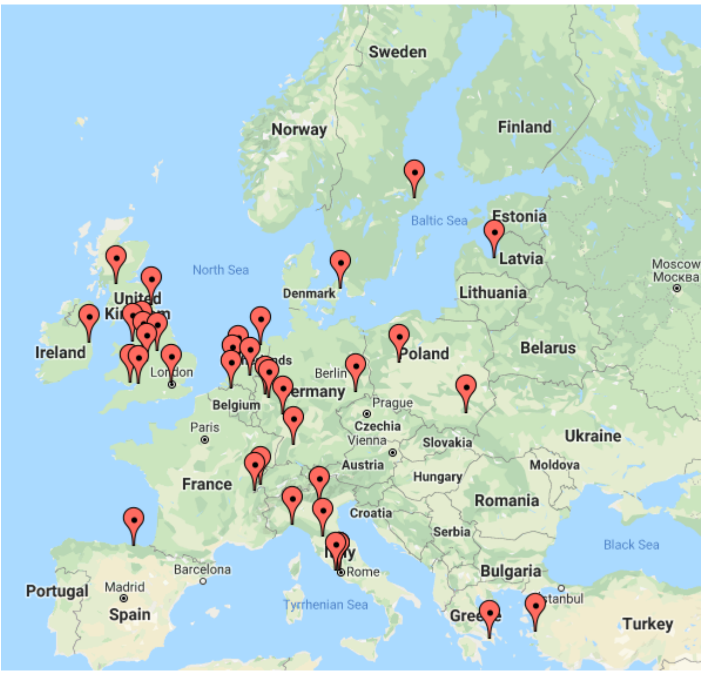

eFigure 2 Map of participating PICUs in Europe

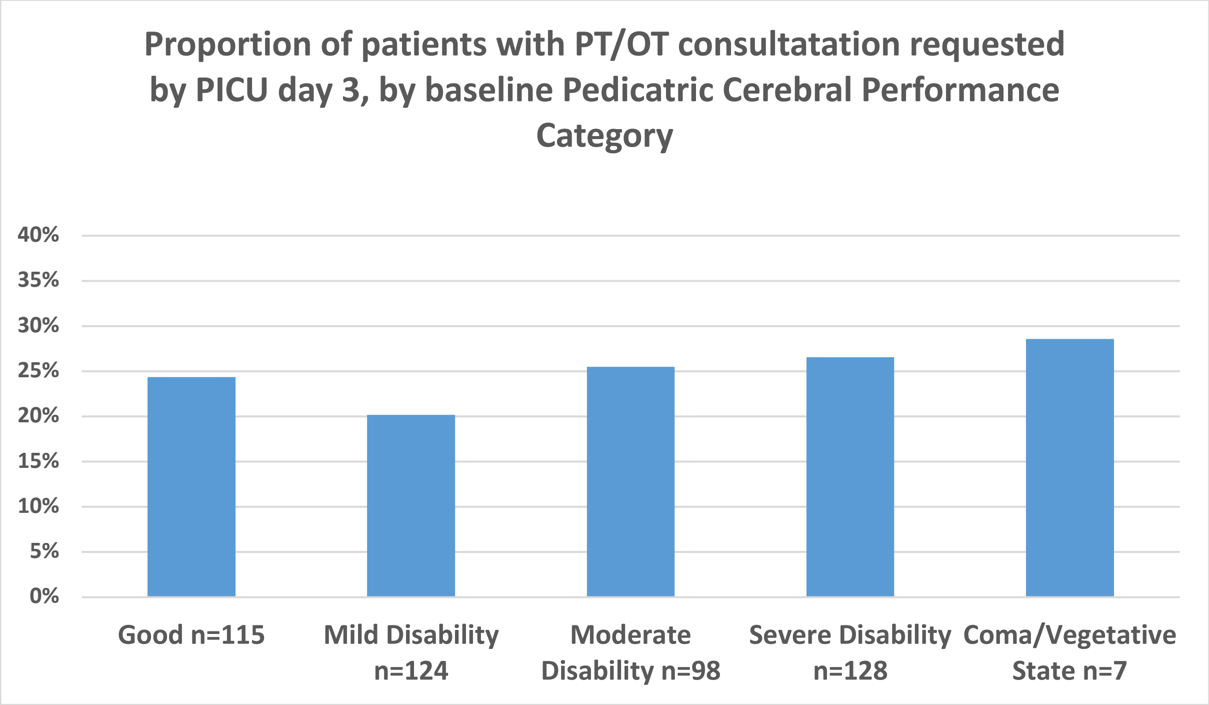

eFigure 3 Proportion of Patients with PT or OT consultation request by Day 3 of PICU admission, by Baseline Pediatric Cerebral Performance Category

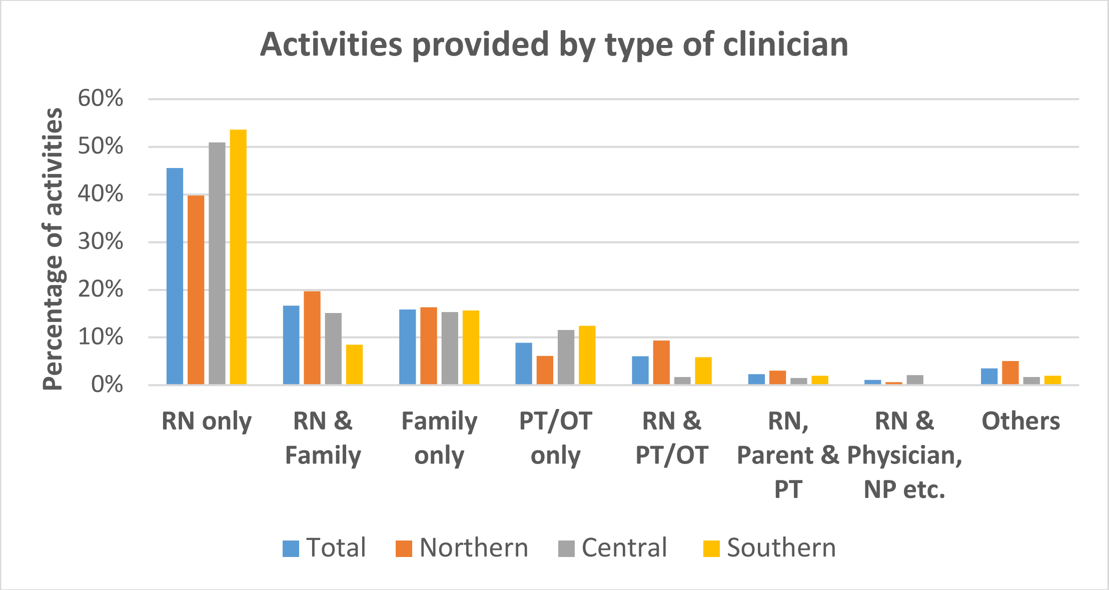

eFigure 4 Activities provided by type of clinician divided by European regions. Northern (Denmark, Ireland, Latvia, the Netherlands, Sweden, and United Kingdom), central (Belgium, Czechia, Germany, Poland, Switzerland), and southern (Greece, Israel, Italy, Spain, and Turkey)

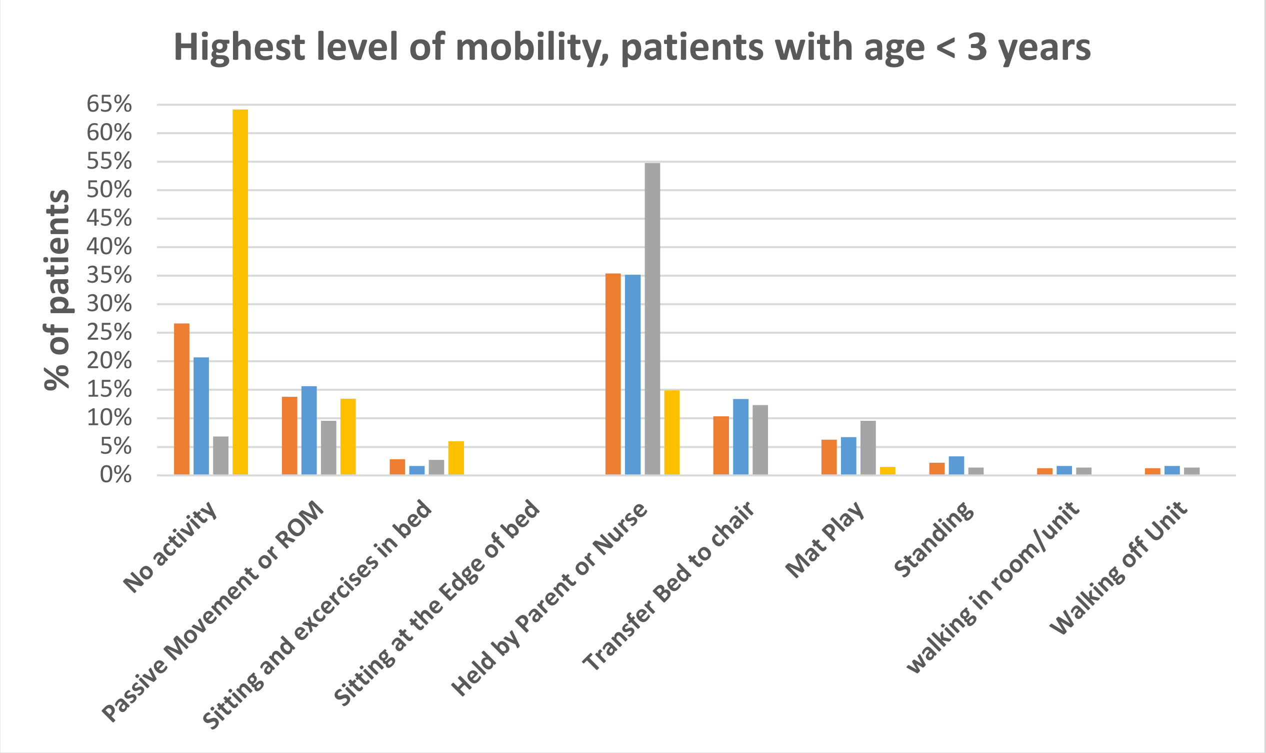

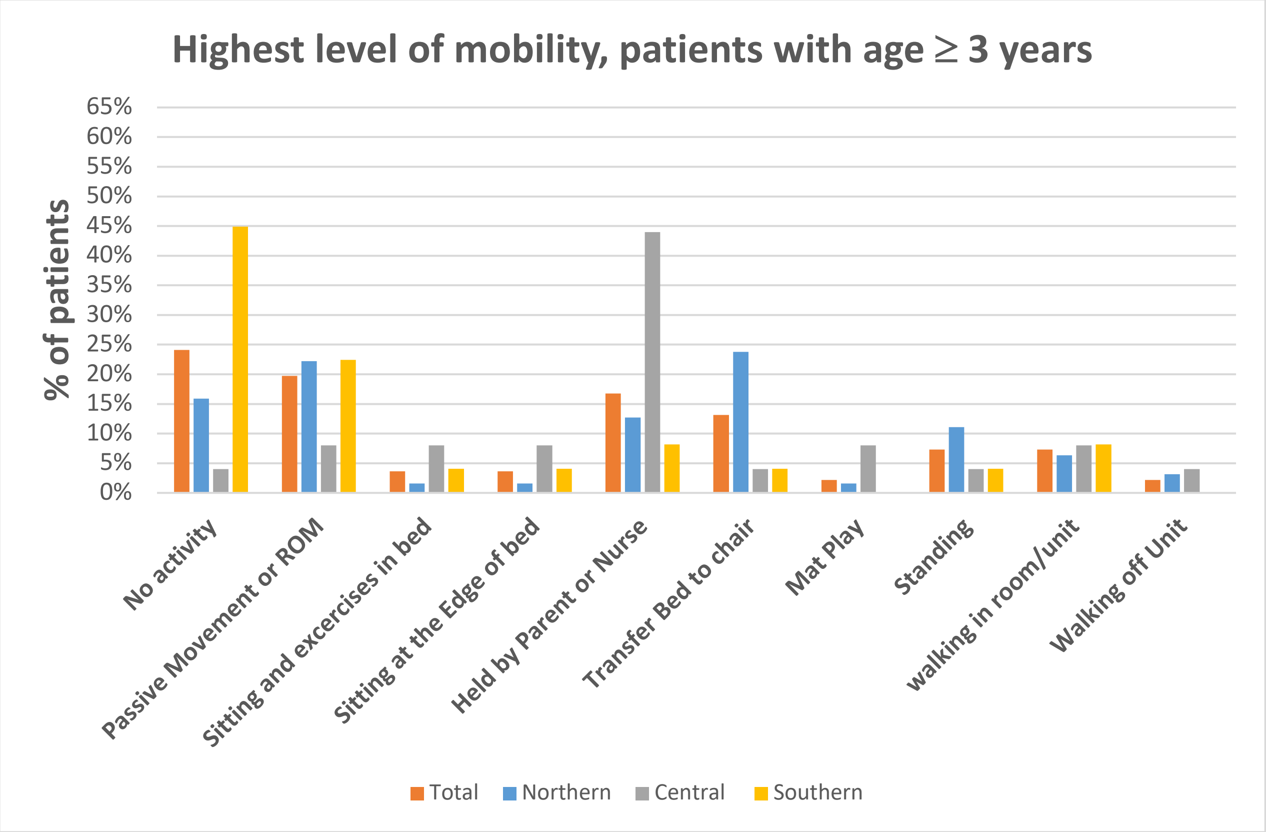

eFigure 5 Highest level of mobility divided by European regions, Northern (Denmark, Ireland, Latvia, the Netherlands, Sweden, and United Kingdom), central (Belgium, Czechia, Germany, Poland, Switzerland), and southern (Greece, Israel, Italy, Spain, and Turkey)
